# Hemi-Nested PCR and RFLP Methodologies for Identifying Blood Meals of the Chagas Disease Vector, *Triatoma infestans*

**DOI:** 10.1371/journal.pone.0074713

**Published:** 2013-09-11

**Authors:** Dawn M. Roellig, Luis A. Gomez-Puerta, Daniel G. Mead, Jesus Pinto, Jenny Ancca-Juarez, Maritza Calderon, Caryn Bern, Robert H. Gilman, Vitaliano A. Cama, the Chagas Disease Workgroup in Arequipa

**Affiliations:** 1 Centers for Disease Control and Prevention, Division of Parasitic Diseases and Malaria, Atlanta, Georgia, United States of America; 2 Universidad Nacional Mayor de San Marcos, Lima, Peru; 3 Southeastern Cooperative Wildlife Disease Study, The University of Georgia, Athens, Georgia, United States of America; 4 Universidad Peruana Cayetano Heredia, Lima Peru; 5 Johns Hopkins University, Bloomberg School of Public Health, Baltimore, Maryland, United States of America; Queensland Institute of Medical Research, Australia

## Abstract

*Trypanosoma cruzi*, the etiologic agent of Chagas disease, is transmitted by hematophagous reduviid bugs within the subfamily Triatominae. These vectors take blood meals from a wide range of hosts, and their feeding behaviors have been used to investigate the ecology and epidemiology of *T. cruzi*. In this study we describe two PCR-based methodologies that amplify a fragment of the 16S mitochondrial rDNA, aimed to improve the identification of blood meal sources for *Triatoma infestans*: a.- Sequence analyses of two heminested PCRs that allow the identification of mammalian and avian species, and b.- restriction fragment length polymorphism (RFLP) analysis from the mammalian PCR to identify and differentiate multi-host blood meals. Findings from both methodologies indicate that host DNA could be detected and the host species identified in samples from laboratory reared and field collected triatomines. The implications of this study are two-fold. First, these methods can be used in areas where the fauna diversity and feeding behavior of the triatomines are unknown. Secondly, the RFLP method led to the identification of multi-host DNA from *T. infestans* gut contents, enhancing the information provided by this assay. These tools are important contributions for ecological and epidemiological studies of vector-borne diseases.

## Introduction

In Latin America, an estimated 8 million people are infected with *Trypanosoma cruzi*, the etiologic agent of Chagas disease, making it one of the most important vector-borne pathogens endemic to the region [1]. Obligate hematophagous triatomine insects in the family *Reduviidae* (subfamily Triatominae) transmit *T. cruzi* and have a wide geographic range stretching from the southernmost tip of South America to northern regions of the United States [2]. While a large number of potential vector species exist, the importance of a vector within a particular region depends greatly on its feeding habits and host preference, as well as the ecology of the area. Reported vertebrate blood meal sources for various *Triatoma* spp. include humans, domestic dogs, rabbits, sheep, opossums, pigs, multiple rodent and bat species, goats, chickens, guinea pigs, lizards and other insects [3]–[9]. In the case of *Triatoma infestans*, one of the most important domiciliary vectors, selective analyses of their blood meals revealed that they feed from domestic dogs, chickens, goats, pigs, cows, cats, domesticated guinea pigs, and humans [3], [4], [10]–[13]

Blood meal analysis is an important tool to determine the vector’s preferred hosts for feeding. Determining the source of the blood meals will help researchers to understand the natural feeding patterns of the vector species and better resolve the epidemiology of Chagas disease in a specific area. Traditional serologic techniques, such as immunodiffusion, double gel diffusion, and precipitin tests have been used previously in blood meal analysis [5]–[7]. However, these assays are limited since they require anti-sera against specific groups or species of animals, which may not be readily available or easily generated. Molecular techniques that amplify high copy-number gene targets, such as mitochondrial genes or repeated elements, may offer a wider range of host identification. Additionally, molecular methods are generally more specific, have better detection limits, and can be more affordable and efficient [3], [4], [8], [9]. For example, traditional polymerase chain reaction methods using species-specific primers, either in single, duplex, or multiplex formats, have been previously used in studies where the suspected hosts are known. However, in cases where the diversity of the fauna and triatomine feeding habits are not fully known, these techniques may provide incomplete data. Amplification of mitochondrial loci has also been used to identify animal species. Fragments of the cytochrome oxidase b locus were previously used for taxonomic and phylogenetic studies [14] while the 12S and 16S rRNA loci were used to identify vertebrate species in forensic studies [15]. Some of these mitochondrial loci have also been used to identify blood meals in vectors [16], [17], and as potential indicators of community based interventions against Chagas disease [18]. These assays relied on DNA sequence analysis for species determination.

The goal of the present study was to develop a robust and simplified method to identify single or multiple blood meal sources of triatomine vectors. To accomplish this, class-specific (mammalia and aves) primers were used in a heminested PCR protocol to amplify a polymorphic region of the 16S rDNA. Sequence analysis of the products allowed the identification of the vertebrate species on which the triatomines had fed. Although, DNA-sequencing is very reliable and becoming more readily available, it is still infrequently available in laboratories where Chagas disease is endemic. Additionally, direct sequencing is not suited for the analysis of two or more co-amplified DNA products. An important alternative is the primary focus of this study, where restriction fragment length polymorphism (RFLP) analysis was developed and used to identify the sources of blood meals for the vectors of *T. cruzi*. These methodologies may prove very valuable for studies to understand the feeding habits of triatomines in endemic foci, especially where the diversity of vector hosts is unknown.

## Materials and Methods

### Ethics Statement

The animal use protocol was approved by the Institutional Animal Care and Use Committee (Comité de Etica y Bienestar Animal) of Universidad Nacional Mayor de San Marcos, School of Veterinary Medicine in Peru, protocol CEBA-0002. All procedures were in accordance to current approved practices by the School of Veterinary Medicine, and in accordance with the AVMA animal welfare principles. The human blood was leftover from a protocol on Chagas disease previously approved by the IRB of Johns Hopkins University that used artificial feeding of triatomines; the consent form from that study allowed the use of leftover material for other research activities. Completely de-identified leftover blood was used in accordance with the signed written consent provided by the study participants. The lead investigator of that study is co-author (RHG) of this manuscript.

### Control samples

Samples from common animal sources of blood for *Triatoma* spp. were collected either through direct collection of blood from laboratory-kept or wild-trapped animals or indirect acquisition in the midgut of laboratory-reared *T. infestans* engorged with blood from specific species.

For collection of blood controls, rats (*Rattus norvegicus*) were anaesthetized with an intramuscular (i.m.) injection of a mixture of 75 mg/kg ketamine (Fort Dodge Laboratories, Inc., Fort Dodge IA, USA) and 5 mg/kg xylazine (Mobay Corporation, Shawneee, KS, USA). Opossums (*Didelphis albiventris*) were anaesthetized with an i.m. injection of a mixture of 10 mg/kg ketamine and 2.0 mg/kg xylazine. Laboratory mice (*Mus musculus*) were restrained but not anesthetized for blood collection.

Laboratory raised *T. infestans* were previously described. Briefly, 3^rd^ and 4^th^ instars were allowed to feed directly on three species (domestic dog, *Canis familiaris;* domestic guinea pig, *Cavia porcellus*; and chicken *Gallus gallus*) or to feed indirectly on anti-coagulated blood from three species of animals (pig, *Sus scrofa*; domestic cat, *Felis catus*; and humans, *Homo sapiens*). Prior to feeding, nymphs were fasted for 4 weeks and maintained at 28°C with a relative humidity of 70–80%. On day 7 post-feeding, *T. infestans* from each animal host group were collected and stored at –20°C until dissection for midgut contents (MC) collection followed by molecular analysis.

For blood samples from rat, opossum, and mouse, the DNA was extracted from 100 µl of blood using the DNeasy Blood and Tissue kit (Qiagen, Inc., Valencia, CA, USA) following the manufacturer’s protocol. The MC of laboratory-fed triatomines were obtained via dissection and stored at −20°C in Tris-EDTA (TE) buffer (10 mM Tris-HCl pH 8.0, 1 mM EDTA) at a 1∶10 ratio. A 100 µL aliquot of this MC was used in DNA extraction methods previously described and stored at −20°C until used in the PCR analysis [19].

### Field samples

A set of 43 randomly selected triatomines were used for further development of the assay. These vectors were collected during a household insecticide spraying campaign conducted by the Arequipa Regional Office of Health in the district of La Joya, Arequipa, Peru. A residual pyrethroid insecticide, solubilized deltamethrin 5% Powder (K-Othrine, Bayer), was applied at 25 mg a.i. per m^2^ to all inhabitable spaces of each home [20]. The captured triatomines were transported the same day to the Universidad Peruana Cayetano Heredia in Arequipa, where species identification, sex (adults), life stage, and infection status were determined. The MC of all triatomines were examined microscopically for the presence of *T. cruzi* metacyclic trypomastigotes [11]. Sterilely-collected aliquots of MC were stored frozen until sent with blind codes to the Centers for Disease Control and Prevention in Atlanta, GA for further analysis. DNA was extracted from MC using the FastDNA Spin Kit for Soil following the manufacturer’s protocol (MP Biomedicals, Irvine, CA) and stored at –80°C until further analysis by PCR.

### Heminested PCR Method

Amplification of the 16S rDNA gene of mammalian and avian groups was accomplished using a universal vertebrate forward primer (VF) (5′ ACC CNT CYM TGT NGC AAA AKR GTG 3′) and group-specific primers for mammalian and avian species. Group-specific primers are as follows: mammalian forward (MF) -5′ CCT GTT TAC CAA AAA CAT CAC 3′, mammalian reverse (MR) -5′ AYT GTC GAT AKG RAC TCT WRA RTA G 3′; avian forward (AF) - 5′ MMC AAG TAT TGA AGG TGA 3′, avian reverse (AR)-5′ CTG ATC CAA CAT CGA GGT CGT 3′ [16]. The primary PCR reaction used the VF primer, and either MF or AF in each reaction. The 50 µL primary reactions contained 0.25 µM each of universal vertebrate forward primer and reverse group-specific primer, 1.5 mM MgCl_2_, 0.25 mM each of the deoxyribonucleoside triphosphates (dNTPs), 0.15 units *Taq* polymerase (Promega Corp.), 5 µL 5X buffer, 2 µL 2% polyvinylpyrrolidone (PVP), and 1 µL extracted DNA as template. The temperature and cycling profile was previously described [21]. The 50 µL secondary reactions contained 0.5 µM each of group-specific forward and reverse primers (MF and MR for mammalian, AF and AR for avian), 1.5 mM MgCl_2_, 0.25 mM each of the dNTPS, 0.15 units *Taq* polymerase, 5 µL 5X buffer, and 2 µL of the primary PCR product as template. The temperature and cycling profile included incubation at 94°C for 2 min 30 sec, followed by 35 cycles as follows: 94°C for 45 sec, 52°C for 1 min, 72°C for 1 min 15 sec, and ended with an indefinite cooling at 4°C. The heminested amplification allows for the detection of small amounts of host DNA in MC, where the blood meals are naturally degraded. Having separate reactions for avian and mammalian species allows for better success in PCR amplification, plus also allows the selective use of the protocol for the discriminating potential reservoirs (mammals) from non-reservoirs (avian). Stringent protocols and controls were used in all PCR assays to prevent and to detect contamination. DNA extraction, amplification and product analysis were performed in separate dedicated laboratory areas. A negative water control was included in each set of extractions and PCR reactions as a contamination control.

### Bi-directional Direct Sequencing

PCR products were purified using Montage PCR filters (Millipore, Billerica, MA) prior to direct sequencing. Amplicons were bi-directionally sequenced using the secondary reaction primers (mammalian F/mammalian R or avian F/avian R) with Big Dye terminator chemistries (Applied Biosystems, Foster City, CA) and run on an ABI 3130x/Genetic Analyzer (Applied Biosystems). Complementary sequences were assembled and verified in ChromasPro 1.5 (Technelysium Pty Ltd). NCBI BLASTN [22] searches were performed on consensus sequences to verify or determine the species of the blood meal source.

### Restriction Length Polymorphism Method

**Figure 1:**
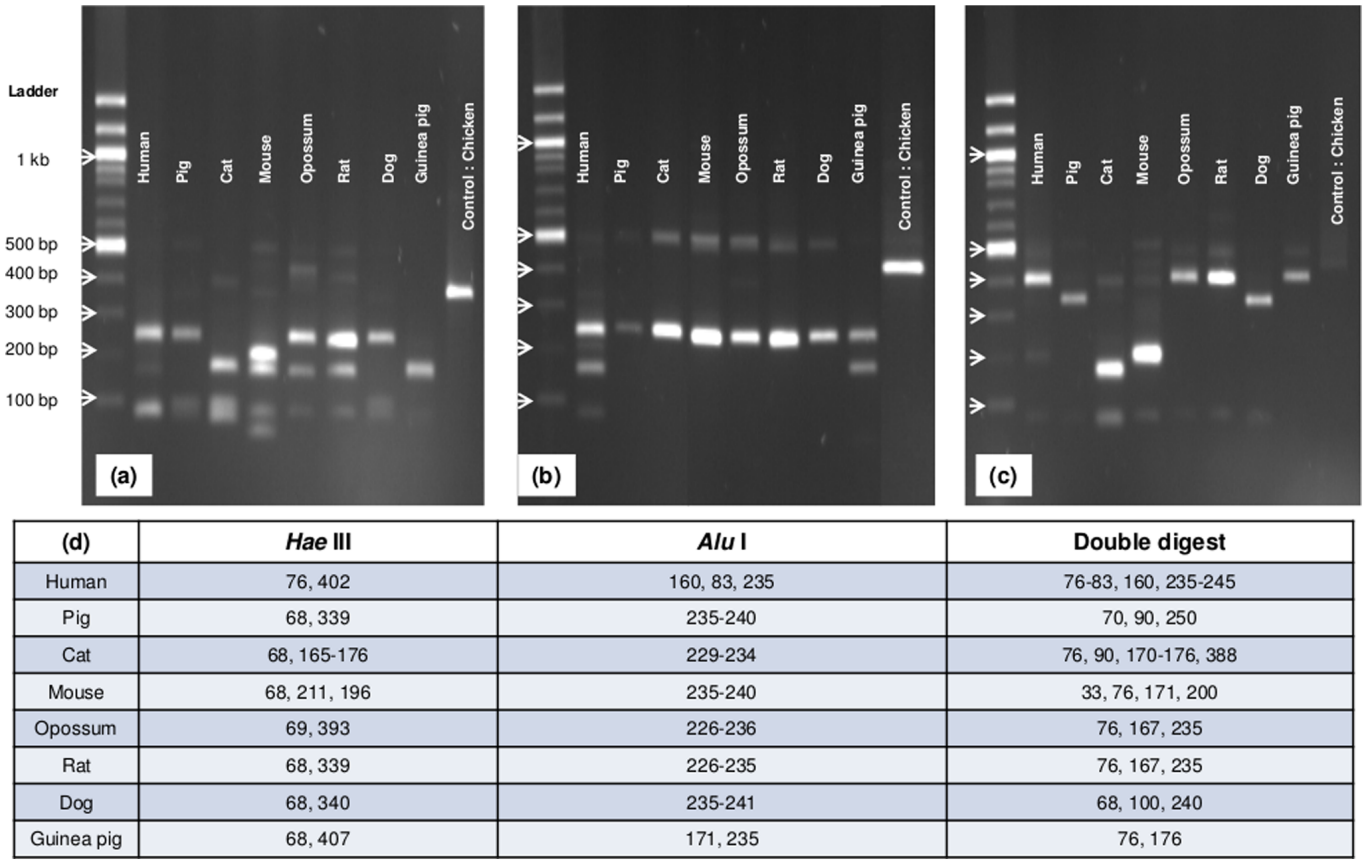
Restriction fragment length polymorphism (RFLP) analysis of 16S rDNA from common Chagas mammalian and avian hosts (laboratory controls), 1.5% agarose gel electrophoresis. (a) Digestion with *Hae* III (b) Digestion with *Alu* I. (c) Simultaneous double digestion with *Hae* III and *Alu* I. (d) Key for fragment sizes following restriction enzyme digestion with *Hae* III, *Alu* I, and simultaneous digestion with both enzymes.

Restriction enzymes were selected based on predicted cuts of the different amplicons and predicted restriction sites determined with ChromasPro 1.5 (Figure 1d). The predicted fragment sizes when digesting with *Hae* III and *Alu* I provided some discriminatory capacity; however the use of both enzymes would yield distinct banding patterns for the analyzed control species. Thus, products were digested with both enzymes.

Heminested PCR products of ∼480 bp were digested with *Hae* III or *Alu* I in a reaction containing: 1 µL enzyme, 2 µL 10X Buffer M (*Hae* III) or L (*Alu* I), 2 µL substrate DNA, and molecular grade water to 20 µL. A double digestion with *Hae* III and *Alu* I contained: 1 µL *Alu* I, 1 µL *Hae* III, 2 µL 10X Buffer L, 2 µL substrate DNA, and molecular grade water to 20 µL. Products were electrophoresed on a 1.5% agarose gel and banding patterns visualized by UV transillumination. Band sizes were estimated using the LabWorks 4.0 software (UVP LLC, Upland, CA).

## Results

### Control samples

Class-specific amplicons of predicted sizes were observed for mammalian and avian species, and confirmation of the reference species was achieved by bidirectional sequence analyses. Distance matrices of the aligned DNA sequences (Figure S1) were constructed using Geneious v. 6.0.3 (www.geneious.com) and Mega v. 5.2.2 (www.megasoftware.net) to show the relative differences between species (Table 1). Digestion of the class-specific amplicons (Figure S2) with *Hae* III resulted in unique banding patterns for cat, mouse, and chicken templates (Figure 1a). Digestion with *Alu* I confirmed the unique, predicted banding patterns for human and guinea pig (Figure 1b). Dog, pig, rat, and opossum could not be readily distinguished by single digestion. To discriminate between pig and dog samples, a double digest with *Alu* I and *Hae* III was successfully used (Figure 1c). Despite additional efforts with enzymes not listed, only rat and opossum could not be distinguished by RFLP analysis; differentiation using sequence analysis was successful for these samples.

**Table 1:** Distance Matrix and Pairwise Distances Between Different Mammalian Species, 16s mitochondrial rDNA locus.

	*Homo sapiens*	*Didelfis marsupialis*	*Suis scrofa*	*Canis familiaris*	*Felis catus*	*Cavia porcellus*	*Mus musculus*	*Rattus rattus*	
Human [DQ834559]		77.0	79.5	79.1	79.9	77.6	78.0	81.2	% Distance matrix (Geneious 6.0.3)
Opossum [DQ283321]	0.257		78.1	78.3	77.4	78.4	79.6	80.6	
Pig [KC469587]	0.216	0.235		85.7	85.3	81.6	81.1	84.7	
Dog [JF342906]	0.210	0.218	0.134		85.8	80.3	80.9	82.4	
Cat [DQ334823]	0.210	0.240	0.136	0.134		79.2	81.5	82.1	
Guinea pig [DQ334847]	0.223	0.217	0.179	0.203	0.206		80.2	82.2	
Mouse [AP013054]	0.237	0.208	0.193	0.184	0.178	0.191		87.4	
Rat [JX105356]	0.191	0.200	0.155	0.163	0.169	0.161	0.113		
	Pairwise distances (Mega 5.2.2)								

Species by scientific common and scientific names [GenBank accession numbers].

### Field samples

**Figure 2:**
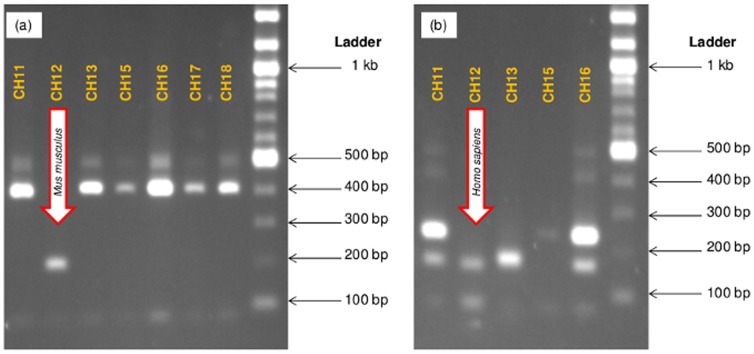
Restriction fragment length polymorphism (RFLP) analysis of 16S rDNA from field-collected *T. infestans* for blood meal analysis. (a) Single digestion with *Hae III* to show sample CH12 with defined pattern for *Mus musculus*. (b) Double digestion with *Hae III* and *Alu I*. Samples CH11 and CH16: rat; CH 13: guinea pig; samples CH15, CH17 and CH18: human. CH12 had DNA from mouse and human (arrows).

The MC of 43 *T. infestans* from Peru were used to determine efficacy of the PCR methodology in conjunction with DNA sequencing or RFLP digestion. DNA was amplified in 30 of 43 field samples: 28 samples had mammalian DNA only and 2 samples had both avian and mammalian DNA. The two avian DNA products were identified as chicken by direct sequence analysis. Additional determination of avian species was not part of the RFLP analysis, as these species are not natural reservoirs of *T. cruzi*. The mammalian species identified by direct sequencing were: guinea pigs (*Cavia porcellus*, n = 17), humans (n = 8), rats (n = 4, *Rattus rattus*), and a domestic cat (*Felis catus*, n = 1). Results from the RFLP analysis of the mammalian amplicons were almost identical to the results from direct sequencing (Table 2). One sample was the exception, as RFLP showed both mouse (*Mus musculus*) and human DNA, while sequencing only identified human DNA (Table 2, Figure 2).

**Table 2:** Blood meal identification of field-collected *Triatoma infestans* by hemi-nested PCR reactions and restriction fragment length polymorphism (RFLP) analysis.

Sample ID	*T. cruzi* status	Avian Identification	Mammal Identification by Sequencing	Mammal Identification by RFLP
CH1	−	n/a	*Cavia porcellus*	*Cavia porcellus*
CH2	+	n/a	*C. porcellus*	*C. porcellus*
CH3	−	n/a	*Homo sapiens*	*Homo sapiens*
CH4	+	n/a	n/a	n/a
CH5	+	n/a	*Felis catus*	*Felis catus*
CH6	−	n/a	*H. sapiens*	*H. sapiens*
CH7	+	n/a	*C. porcellus*	*C. porcellus*
CH8	+	n/a	n/a	n/a
CH9	−	n/a	*C. porcellus*	*C. porcellus*
CH10	−	n/a	n/a	n/a
CH11	+	n/a	*Rattus rattus*	*Rattus rattus*
CH12	−	*Gallus gallus*	*H. sapiens*	*H. sapiens* + *Mus musculus*
CH13	−	n/a	*C. porcellus*	*C. porcellus*
CH14	−	n/a	n/a	n/a
CH15	−	n/a	*H. sapiens*	*H. sapiens*
CH16	+	n/a	*R. rattus*	*R. rattus*
CH17	−	n/a	*H. sapiens*	*H. sapiens*
CH18	+	n/a	*H. sapiens*	*H. sapiens*
CH19	+	n/a	*H. sapiens*	*H. sapiens*
CH20	−	*G. gallus*	*C. porcellus*	*C. porcellus*
CH21	+	n/a	*C. porcellus*	*C. porcellus*
CH22	+	n/a	n/a	n/a
CH23	−	n/a	*H. sapiens*	*H. sapiens*
CH24	+	n/a	*C. porcellus*	*C. porcellus*
CH25	+	n/a	n/a	n/a
CH26	+	n/a	*C. porcellus*	*C. porcellus*
CH27	+	n/a	*R. rattus*	*R. rattus*
CH28	+	n/a	n/a	n/a
CH29	+	n/a	n/a	n/a
CH30	+	n/a	*R. rattus*	*R. rattus*
CH31	+	n/a	*C. porcellus*	*C. porcellus*
CH32	−	n/a	*C. porcellus*	*C. porcellus*
CH33	+	n/a	n/a	n/a
CH34	−	n/a	n/a	n/a
CH35	+	n/a	*C. porcellus*	*C. porcellus*
CH36	+	n/a	*C. porcellus*	*C. porcellus*
CH37	−	n/a	n/a	n/a
CH38	+	n/a	n/a	n/a
CH39	+	n/a	*C. porcellus*	*C. porcellus*
CH40	+	n/a	*C. porcellus*	*C. porcellus*
CH41	+	n/a	*C. porcellus*	*C. porcellus*
CH42	−	n/a	*C. porcellus*	*C. porcellus*
CH43	+	n/a	n/a	n/a

+  =  positive for *T. cruzi* by microscopy.

−  =  negative for *T. cruzi* by microscopy.

n/a  =  not applicable, samples were negative for animal DNA by PCR.

The 43 MC were also analyzed for the microscopic presence of *T. cruzi* epimastigotes with 27 samples being positive. Among the *T. cruzi* positive, blood meal sources were identified for 18 triatomines; these included guinea pigs (n = 11), rats (n = 4), humans (n = 2), and domestic cat (n = 1).

## Discussion

Determining host preference and feeding patterns is an important tool in epidemiologic studies of vector-borne diseases. In this study, we provide a robust sequence-based method and an alternative methodology for differentiating the species of common vertebrate hosts of Chagas disease vectors, which also allows for the simultaneous identification of blood from more than one mammalian host.

PCR-based molecular techniques have allowed for sensitive and specific identification of blood meal hosts compared to the previously used immunologic assays [23]. For Chagas disease vectors, several methodologies have been described using species-specific oligonucleotides to determine feeding preferences [3], [4], [8], [19]. The disadvantage of such techniques is the need for *a priori* knowledge of potential hosts in the area.

The use of universal primers has allowed researchers to identify a more diverse range of hosts. DNA sequencing of the cytochrome oxidase B gene (cytB) using primers previously developed [14] was used to barcode the sources of blood meals for *Culex tarsalis*, with an overall host identification in 61% of samples, and 72% detection rate among fully engorged mosquitoes [24]. PCR amplification and DNA sequencing of the 12S and 16S rRNA mitochondrial loci were successfully developed for identification of vertebrate species in forensic investigations, and used to determined that a broken bone found in a construction site belonged to a pig [15]. These protocols were also used in studies of Chagas in the United States, where cloned PCR products of the cytB [14]and 12S mitochondrial [15] genes were used to analyze the blood meals of 13 vectors. Date from the 12S locus allowed the identification of DNA in four vectors: two had human and dog DNA, one had dog DNA alone, and one with dog and pig DNA. Given the animal diversity in the study site, it was concluded that the pig DNA could likely be from javelina (*Tauassu tajacu*), and the dog DNA could also be from either coyotes (*Canis latrans*) or wolves (*Canis lupus*). Recent studies have described new primers to amplify the 12S locus; this new protocol identified blood meals in 88 of 109 vectors using real time PCR amplification followed by DNA sequencing [17].

The development of an assay that can also detect vertebrate DNA from the taxonomical classes aves and mammalia targeting a species discriminatory region of the 16S rDNA expands the current set of tools for species identification in blood meals from vectors of disease. The heminested PCR protocol described herein is a modification from a methodology used to detect blood meal preferences of *Lutzomyia shannoni* in vesicular stomatitis studies [21]. Our current methodology offers the advantage of detecting both avian and mammalian species, which can be sequenced and compared to references already deposited in GenBank for species identification.

Another and perhaps greater contribution of our methods is the potential to identify one or more species without DNA sequencing. It is known that blood meals from *Triatoma* spp. may contain DNA from more than one vertebrate species [4], [14], [25], [26]. Thus, an important consideration for blood meal analysis is the ability to detect blood from more than one host in a single triatomine. PCR can amplify DNA fragments from multiple hosts in a single sample, but sequencing reactions would either selectively amplify the most abundant product or result in overlapping base reads, confounding results. In order to resolve this, DNA sequencing alone was complemented and improved by developing an RFLP technique. The use of a single and double digestion with commercially available restriction enzymes allowed for the identification of known *T. cruzi* reservoirs as well as other hosts where the vectors feed on. The RFLP has the potential to identify multiple hosts at once because of the unique banding patterns described in the current study.

We evaluated the efficacy of the described methodologies with field-collected triatomines. Those *T. infestans* were previously collected in a study conducted in Arequipa, Peru, in an urban area where Chagas disease is endemic. The heminested PCRs for both avian and mammalian species successfully amplified host DNA that could be speciated by sequencing analysis. Concordant results were obtained with the RFLP analysis, indicating the accuracy of the newly developed methodology. These findings were also in line with previous reports that guinea pigs, chickens, cows and sheep in these areas lived in close proximity to humans [27], and that the presence of dogs and cats were significantly associated with increased risk for *T. cruzi* infections [28]. Our study detected DNA from guinea pigs, humans and cats in 17, 8 and 1 of the samples with positive PCR amplification, respectively. These findings highlight the importance of identification of host by blood meal analysis, as it is know that there is a 2.4 fold increase in the density of triatomines in households that have guinea pigs, and that the presence of dogs and cats in the sleeping area resulted in about 5.2 more insects per room when compared to rooms without animals [20]. In addition, the advantage of using the RFLP analysis for multi-host detection was highlighted by identifying both human and mouse DNA in the MC from a single triatomine. Digestion with *Hae III* alone resulted in a mouse identification compared to the double digest that resulted in blood meal identification as human. This technique allowed for the rapid and inexpensive identification of both hosts in comparison to the common practice of running several species-specific PCR reactions or the use of DNA sequencing of PCR amplified products [3],[4],[26].

The heminested PCR and RFLP analyses reported in this study provide a rapid, inexpensive, and easy tool for identifying blood meal profiles of disease vectors. It could be applied in studies or programs that use ecological-based approaches for public health improvement, since blood meal analyses can be important outcomes to evaluate the effects of interventions to control vectors of Chagas disease [18]. While the goal for the current study was to develop and evaluate improved methodologies for blood meal analyses from Chagas disease vectors, the use of both of these tools for other vector-borne diseases may prove advantageous. Further validations with samples from more diverse animal species, MC from rural areas and other vector species must be conducted to fully appreciate the usefulness of the described techniques in epidemiological studies.

## Supporting Information

Figure S1 DNA sequence alignment of the 16s mitochondrial rRNA locus amplified in this study.
(PDF)

Figure S2 Predicted digestion of 16S rRNA from common *T. cruzi* and *T. infestans* mammalian hosts using *Hae* III (a), *Alu* I (b), or double digestion with both *Hae* III and *Alu* I (c) as restriction enzymes in a restriction fragment length polymorphism (RFLP).
(JPG)
