# Supplementary material for: Hemi-Nested PCR and RFLP Methodologies for Identifying Blood Meals of the Chagas Disease Vector, Triatoma infestans
Source: PLoS One. 2013 Sep 11;8(9):e74713. doi: 10.1371/journal.pone.0074713 (PMC3770599; doi:10.1371/journal.pone.0074713)
Supplement: Figure S1 — DNA sequence alignment of the 16s mitochondrial rRNA locus amplified in this study. (PDF) [file pone.0074713.s001.pdf]

Species_GenBank Accession #	
Human_DQ834559.	CCTGTTTACCAAAAACATCACCTCTAGCATCACCAGTATTAGAGGCACCG
Opossum_DQ283321.	CCTGTTTACCAAAAACATCACCTCTAGCATTCCAAGTATTAGAGGCACTG
Pig_KC469587.	CCTGTTTACCAAAAACATCACCTCTAGCATTACTAGTATTAGAGGCAATG
Dog_JF342906.	CCTGTTTACCAAAAACATCACCTCCAGCATTTCTAGTATTGGAGGCACTG
Cat_DQ334823.	CCTGTTTACCAAAAACATCACCTCTAGCATTTCTAGTATTAGAGGCACTG
Guinea_pig_DQ334847.	CCTGTTTACCAAAAACATCACCTCTAGCATACTTAGTATTAGAGGCACTG
Mouse_AP013054.	CCTGTTTACCAAAAACATCACCTCTAGCATTACAAGTATTAGAGGCACTG
Rat_JX105356.	CCTGTTTACCAAAAACATCACCTCTAGCATAACAAGTATTAGAGGCATTG

Human_DQ834559.	CCTGCCCAGTG-ACACATGTTTAACGGCCGCGGTACCCTAACCGTGCAAA
Opossum_DQ283321.	CCTGCCCAGTGAATAAACTTTTAACGGCCGCGGTATCCTGACCGTGCAAA
Pig_KC469587.	CCTGCCCAGTG-ACACCAGTTTAACGGCCGCGGTATTCTGACCGTGCAAA
Dog_JF342906.	CCTGCCCGGTG-ACACTTGTTTAACGGCCGCGGTATCCTGACCGTGCAAA
Cat_DQ334823.	CCTGCCCGGTG-ACGCTAGTTAAACGGCCGCGGTATCCTGACCGTGCAAA
Guinea_pig_DQ334847.	CCTGCCCAGTG-ACATTCGTTCAACGGCCGCGGTATCCTGACCGTGCAAA
Mouse_AP013054.	CCTGCCCAGTG-ACTAAAGTTTAACGGCCGCGGTATCCTGACCGTGCAAA
Rat_JX105356.	CCTGCCCAGTG-ACTAAAGTTAAACGGCCGCGGTATCCTGACCGTGCAAA

Human_DQ834559.	GGTAGCATAATCACTTGTTCCTTAAATAGGGACCTGTATGAATGGCTCCA
Opossum_DQ283321.	GGTAGCATAATCACTTGTCTCCTAAATAGGGACTTGTATGAATGGCATAA
Pig_KC469587.	GGTAGCATAATCACTTGTTCTCCAAATAAGGACTTGTATGAATGGCCACA
Dog_JF342906.	GGTAGCATAATCATTTGTTCTCTAAATAGGGACTTGTATGAATGGCCACA
Cat_DQ334823.	GGTAGCATAATCATTTGTTCCCTAAATAGGGACTTGTATGAACGGCCACA
Guinea_pig_DQ334847.	GGTAGCATAATCACTTGTTCTTTAAATAAGGACTAGTATGAATGGCAAGA
Mouse_AP013054.	GGTAGCATAATCACTTGTTCCTTAATTAGGGACTAGCATGAACGGCTAAA
Rat_JX105356.	GGTAGCATAATCACTTGTTCCTTAATTAGGGACTAGAATGAATGGCTAAA

Human_DQ834559.	CGAGGGTTCAGCTGTCTCTTACTTTTAACCAGTGAAATTGACCTGCCCGT
Opossum_DQ283321.	CGAGGGTTCAACTGTCTCTTCTTCTTAATCAATGAAATTGACCTACCCGT
Pig_KC469587.	CGAGGGTTTTACTGTCTCTTACTTCCAATCAGTGAAATTAACCTTCCCGT
Dog_JF342906.	CGAGGGTTTAACTGTCTCTTACTCCCAATCAGTGAAATTGACCTTCCCGT
Cat_DQ334823.	CGAGGGCTTTACTGTCTCTTACTTCCAATCCGTGAAATTGACCTTCCCGT
Guinea_pig_DQ334847.	CGAGGGTTTATCTGTCTCTTGCTTTACGTCAGTGAAATTGATCTTCCCGT
Mouse_AP013054.	CGAGGGTCCAACTGTCTCTTATCTTTAATCAGTGAAATTGACCTTTCAGT
Rat_JX105356.	CGAGGGTTCAACTGTCTCTTACTTTCAATCAGTGAAATTGACCTTCCAGT

```
Human_DQ834559.       G A A G A G G C G G G C A T G A C A C A G C A A G A C G A G A A G A C C C T A T G G A G C T T T A A
Opossum_DQ283321.     G C A G A G G C G G G T A T A C T A A T A T A A G A C G A G A A G A C C C T G T G G A G C T T A A G
Pig_KC469587.         G A A G A G G C G G G A A T A A A A A A A T A A G A C G A G A A G A C C C T A T G G A G C T T T A A
Dog_JF342906.         G A A G A G G C G G G A A T A C C A C A A T A A G A C G A G A A G A C C C T A T G G A G C T T T A A
Cat_DQ334823.         G A A G A G G C G G G A A T A T A A T A A T A A G A C G A G A A G A C C C T A T G G A G C T T T A A
Guinea_pig_DQ334847.  G A A G A A G C G G G A A T A A T A T A A T A A G A C G A G A A G A C C C T A T G G A G C T T T A A
Mouse_AP013054.       G A A G A G G C T G A A A T A T A A T A A T A A G A C G A G A A G A C C C T A T G G A G C T T A A A
Rat_JX105356.         G A A G A G G C T G G A A T C T C C C A A T A A G A C G A G A A G A C C C T A T G G A G C T T T A A

Human_DQ834559.       T T T A T T A A T G C A A A C A G T A C C T - - - A A C A A A C C C A C A G G T C C T A A A C T A C
Opossum_DQ283321.     A C T A A T A A C T T A A A T A A A A C T A - - - A C A C A A A C C C T A G G G A A T A A C A T T A
Pig_KC469587.         T T A A C T A T T C C A A A A G T T A A A C A - - A C T C A A C C A C A A A G G G A T A A A A C A T
Dog_JF342906.         T T A A C T A A C C C A A A C T T A T G G A T A C T A G A T A C C T A C A A G G C A T A A C A T A A
Cat_DQ334823.         T T A A C C G A C C C A A A G A G A C C A T A T G A A C C A A C C G A C A G G A A C A A C A A A C C
Guinea_pig_DQ334847.  T T T A T T A G C T T A C T T A A C C A A T - - - T T T T A A C C C A T A A G G G C - A T A A C A C
Mouse_AP013054.       T T A T A T A A C T T A T C T A T T T A A T T - - T A T T A A A C C T A A T G G C C C A A A A A C T
Rat_JX105356.         T T T A C T A G T T C A A C T T A T A T A A - - - A A A C A A C C T A - A T G G G C T A A A A C A A

Human_DQ834559.       - - - - - - - C A A A C C T G C A T T A A - A A A T T T C G G T T G G G G C G A C C T C G G A G C A
Opossum_DQ283321.     - - - - - - - T T A T C C T T A A G T T A T A T T C T T T G G T T G G G G T G A C C T C G G A G A A
Pig_KC469587.         A A C - - - - T T A A C A T G G A C T A G - C A A T T T C G G T T G G G G T G A C C T C G G A G T A
Dog_JF342906.         C A C C A - - T T A T T A T G A G T T A G - C A A T T T A G G T T G G G G T G A C C T C G G A A T A
Cat_DQ334823.         - - - - - - - T C T A T A T G G G C C G G - C A A T T T A G G T T G G G G T G A C C T C G G A G A A
Guinea_pig_DQ334847.  A T A C A C T T A A A C C T A A G C T A A - T A A T T T C G G T T G G G G T G A C C T C G G A G T A
Mouse_AP013054.       - - - - - - - A T A G T A T A A G T T T G - A A A T T T C G G T T G G G G C G A C C T C G G A G A A
Rat_JX105356.         A A - - - - - T A A A T A T G A A C T A A A A A A T T T C G G T T G G G G T G A C C T C G G A G A A

Human_DQ834559.       G A A C C C A A C T C C G A G C A G T A C A T G C T A A G A C T T C A C C A G T C - A A A G C G A
Opossum_DQ283321.     T A A A A A A A C C T C C G A A T G A T A T - A A C C T A G A T C A - A C C A A T C - C A A G T G C
Pig_KC469587.         C A A A A A A C C C T C C G A G T G A T T T T A A T C T A G A C A A - A C C A G T C - A A A A T A A
Dog_JF342906.         T A A A A A A A C T C C C G A G T G A T T A A A A T T T A G A C C C - A C A A G T C - A A A A T A -
Cat_DQ334823.         C A A A A C A A C C T C C G A G T G A T T T A A A T C T A G A C T A - A C C A G T C G A A A G T A -
Guinea_pig_DQ334847.  C A A T A A A A C C T C C G A A T G A T A T T A G C C T A G A T C C A A C A A A T C - G A A G T - -
Mouse_AP013054.       T A A A A A A T C C T C C G A A T G A T T A T A A C C T A G A C T T - A C A A G T C - A A A G T A A
Rat_JX105356.         T A A A A A A T C C T C C G A A T G A T T T T A A C C T A G A C T C - A C A A G T C - A A A G T A A
```

```
Human_DQ834559.        A C - T A C T A T A C T C A A T T G A T C C A A T A A - - - C T T G A C C A A C G G A A C A A G T T
Opossum_DQ283321.      A C - A A A A G C C A G T A A T T G A C C C A A A T A - - - - T T G A T C A A C G G A A C A A G T T
Pig_KC469587.          C C A T A A C A T C A C T T A T T G A T C C A A A A T - - - T T T G A T C A A C G G A A C A A G T T
Dog_JF342906.          - - - C A A C A T C A C T T A T T G A T C C A A T A A - T T T T T G A T C A A C G G A A C A A G T T
Cat_DQ334823.          - - - C T A C A T C A C T T A T T G A T C C A A A A A - - C C T T G A T C A A C G G A A C A A G T T
Guinea_pig_DQ334847.   - - - T T A A A T C A C C A A T T G A C C C A A A T T C C T T T T G A T C A A C G G A C C A A G T T
Mouse_AP013054.        A A T C A A C A T A T C T T A T T G A C C C A G A T A T A T T T T G A T C A A C G G A C C A A G T T
Rat_JX105356.          T A C T A A T A T - - C T T A T T G A C C C A A T T A - - - - T T G A T C A A C G G A C C A A G T T

Human_DQ834559.        A C C C T A G G G A T A A C A G C G C A A T C C T A T T C T A G A G T C C A T A T C A A C A A - - T
Opossum_DQ283321.      A C C C C A G G G A T A A C A G C G C A A T C C T A T T T A A G A G C C C A T A T C G A A A A T C T
Pig_KC469587.          A C C C T A G G G A T A A C A G C G C A A T C C T G T T C T A G A G T T C C T A T C G A C A A - - T
Dog_JF342906.          A C C C T A G G G A T A A C A G C G C A A T C C T A T T C A A G A G T C C A T A T C G A C A A - - T
Cat_DQ334823.          A C C C T A G G G A T A A C A G C G C A A T C C T A T T T C A G A G T C C A T A T C G A C A A - - T
Guinea_pig_DQ334847.   A C C C T A G G G A T A A C A G C G C A A T C C T A T T C T A G A G T T C A T A T C G A C A A - - T
Mouse_AP013054.        A C C C T A G G G A T A A C A G C G C A A T C C T A T T T A A G A G T T C A T A T C G A C A A T - T
Rat_JX105356.          A C C C T A G G G A T A A C A G C G C A A T C C T A T T T A A G A G T T C A T A T C G A C A A T - T

Human_DQ834559.        A G G G T T T A C G A C C T C G A T G T T G G A T C A G G A C A T C C C G A T G G
Opossum_DQ283321.      A G G G T T T A C G A C C T C G A T G T T G G A T C A G G A C A T C C T A A T G G
Pig_KC469587.          A G G G T T T A C G A C C T C G A T G T T G G A T C A G G A C A C C C A A A T G G
Dog_JF342906.          A G G G T T T A C G A C C T C G A T G T T G G A T C A G G A C A T C C T A A T G G
Cat_DQ334823.          A G G G T T T A C G A C C T C G A T G T T G G A T C A G G A C A T C C C G A T G G
Guinea_pig_DQ334847.   G G G G T T T A C G A C C T C G A T G T T G G A T C A G G A C A T C C T A A T G G
Mouse_AP013054.        A G G G T T T A C G A C C T C G A T G T T G G A T C A G G A C A T C C C A A T G G
Rat_JX105356.          A G G G T T T A C G A C C T C G A T G T T G G A T C A G G A C A T C C C A A T G G
```
