# Supplementary figures and images for: Hemi-Nested PCR and RFLP Methodologies for Identifying Blood Meals of the Chagas Disease Vector, Triatoma infestans
Source: PLoS One. 2013 Sep 11;8(9):e74713. doi: 10.1371/journal.pone.0074713 (PMC3770599; doi:10.1371/journal.pone.0074713)

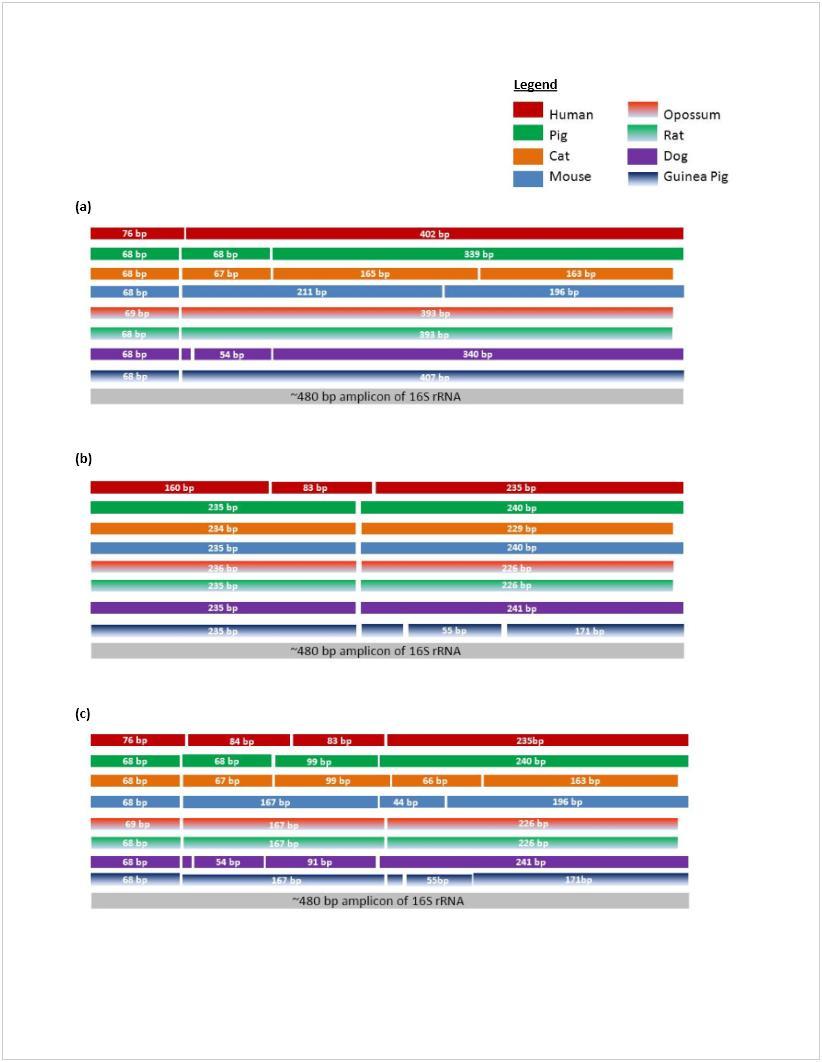

Supplement: Figure S2 — Predicted digestion of 16S rRNA from common T. cruzi and T. infestans mammalian hosts using Hae III (a), Alu I (b), or double digestion with both Hae III and Alu I (c) as restriction enzymes in a restriction fragment length polymorphism (RFLP). (JPG) [file pone.0074713.s002.jpg]
